# Epidemiological and histopathological profile of malignant melanoma in Malawi

**DOI:** 10.1186/s12907-019-0087-6

**Published:** 2019-04-02

**Authors:** Maurice Mulenga, Nathan D. Montgomery, Maganizo Chagomerana, Tenganawo Mzumala, Tamiwe Tomoka, Coxcilly Kampani, Yuri Fedoriw, Satish Gopal, Lisbet Sviland

**Affiliations:** 1 Kamuzu Central Hospital, Lilongwe, Malawi; 2 Haukeland University Hospital, Helse Bergen, Norway; 3 Malawi UNC Project-Malawi, Lilongwe, Malawi; 4 Department of Pathology and Laboratory Medicine, University of North Carolina, Chapel Hill, USA

## Abstract

**Background:**

Studies on malignant melanoma have largely focused on Caucasian populations due to higher incidence in lighter-skinned individuals. While there is a well developed body of literature describing melanoma in African-Americans, much less is known about melanoma in black Africans. Prior reports have suggested that it is reportedly extremely rare in black Africans who are considered to mostly have the acral lentiginous subtype. However, an accurate understanding of melanoma in this part of the world is hindered by the very limited nature of prior publications. The aim of this study was to determine the epidemiological profile, anatomical distribution and histopathological features of melanoma presenting in Africans at a tertiary referral hospital in Malawi.

**Methods:**

This is a retrospective study that characterized melanoma cases diagnosed from January 2012 to December 2017, at a cancer referral centre in Malawi. All confirmed, malignant melanoma cases during the study period were retrieved. Data abstracted included age, sex, anatomic site and whether it was a primary or metastatic site. Breslow thickness in millimetres, Clark level of invasion, presence of ulceration and melanoma subtype were also evaluated.

**Results:**

One hundred thirty-two cases were included in the study, 81 (61%) were female and 26 (20%) were from a metastatic site. The mean age was 57 years (sd = 15) with the majority in the age group 60–69 years. Males presented at an older age than females. Ninety five percent of cutaneous melanomas were located on acral sites, most commonly the foot (87%) and the most common histopathological subtype was acral lentiginous. Eighty four percent presented with a Breslow thickness over 4 mm (median 9 mm).

**Conclusion:**

Our study shows that malignant melanoma occurs in black people in Malawi and may be an under-appreciated malignancy. While long term clinical follow-up was not available, most patients presented at late stages of the disease, supporting a poor prognosis. These results suggest that increased awareness of melanoma in black Africans and earlier intervention may have meaningful impacts on outcomes and survival.

## Background

GLOBOCAN 2018 estimates for worldwide cancer burden suggest that there are nearly 300,000 new cases of melanoma annually, resulting in more than 60,000 deaths [1]. However studies of malignant melanoma largely include Caucasian populations, in part because of higher incidence in lighter-skinned individuals [2]. In contrast, melanoma is reportedly rare among black Africans. Studies in Nigeria, Togo and South Africa identified only 15, 63 and 185 cases respectively over periods of 11, 20 and 15 years [3–5]. According to World Health Organisation (WHO), [6] acral lentiginous melanoma (ALM) is considered the most common subtype among Africans, however this conclusion is largely based on studies of the African-Americans and black South Africans [7–9]. For instance, studies performed in the United States showed that sole or palm (44%), lower extremities (63%) and foot were the common sites of melanoma in black Americans [10–12] and in a South African study, ALM was the only type found in black Africans. In their cohort, no cases occurred above the wrist or ankle [5]. Although genetic predisposition has been implicated in the predominance of ALM in patients of African ethnicity [9], African-Americans and black Africans are not genetically identical [13]. The aim of this study was to determine the epidemiological profile, anatomical distribution and histopathological details of melanoma at a tertiary referral hospital in Malawi.

## Methods

A retrospective study was conducted to characterize melanoma cases diagnosed from January 2012 to December 2017 at Kamuzu Central Hospital (KCH), a cancer referral centre for approximately nine million people in Malawi. The histopathology laboratory of KCH opened in July 2011 and receives specimens from 2 central hospitals, 15 government district hospitals and more than 20 private hospitals and clinics. All confirmed, malignant melanoma cases during the study period were retrieved in the pathology database. Data abstracted included age, sex, anatomic site and whether it was a primary or metastatic site. Anatomic sites were grouped as head and neck (including face), trunk, extremities (lower and upper), acral, and unknown. We also assessed Breslow thickness in millimetres, Clark level of invasion, and presence of ulceration. Cases were also further classified as nodular melanoma (NMM), superficial spreading melanoma (SSM), acral lentiginous melanoma (ALM), lentigo maligna melanoma (LMM) and unclassified. Breslow thickness was measured from the granular layer or, when present, the ulcer base to the deepest extent of invasion by tumour cells. Breslow thickness was then categorized into four groups (≤1.00 mm, 1.01–2.0 mm, 2.01–4.0 mm, > 4.0 mm for pT1, pT2, pT3 and pT4 respectively) to determine the T stage as per the UICC7 pTNM staging. The level of invasion was determined as defined by Dr. Wallace Clark [13] (level I—confined to the epidermis, level II—invasion of the papillary dermis, level III—invasion to the papillary/reticular interface, level IV—invasion to the reticular dermis, level V—invasion to the subcutaneous tissue).

We used proportions and medians to summarize the distribution of categorical variables and continuous variables, respectively. Fisher’s exact test was used to assess the association between gender and acral lentiginous melanoma. All analyses were performed using Stata software, version 14.1 (StataCorp LP, College Station, Texas, USA). Permission to conduct this analysis was approved under the approval of the pathology databases. The need for informed consent was exempted from institutional review board due to the nature of the study, which involved a retrospective analysis of routinely collected data.

## Results

### Clinical characteristics

Between 2012 to 2017, there were 132 cases of invasive and in-situ melanoma diagnosed at Kamuzu Central Hospital (Lilongwe, Malawi) and included in the study, of which 81 (61%) were from females.

**Fig. 1:**
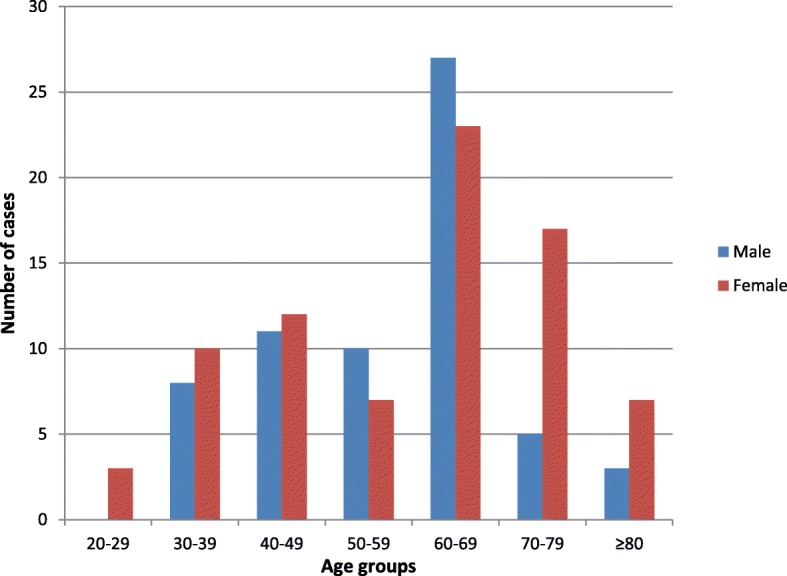
Age group distribution among the melanoma cases at Kamuzu Central Hospital, Malawi (2012–2017). Legend: The number of melanoma cases diagnosed at Kamuzu Central Hospital Pathology Laboratory between 2012 and 2017 is shown. Melanomas diagnosed in males and females are shown separately, with grouping by decade of life of diagnosis

The mean age among cases was 57 years with a standard deviation of 15. The age range was 26 to 91 years and the cases peaked in the age group 60-69 years (Fig. 1). Male cases were older (mean age 62 years) than females (mean age 53 years). This was statistically significant, *p* = 0.0211.

The primary site was known for 106 cases, including 103 cutaneous melanomas and 3 mucosal melanomas. 98 cases (95%) of cutaneous melanomas were located on acral sites, and 90 cases (87%) were on the foot. Only 8 cases presented on the hands, with 7 of 8 occurring in females (Table 1).

**Table 1:** Melanoma distribution by anatomical site and gender at Kamuzu Central Hospital, Malawi (2012–2017)

ANATOMICAL SITE	MALE	FEMALE	TOTAL
Head and neck	2	2	4
Trunk	0	1	1
Upper extremity (excluding acral sites)	0	1	1
Lower extremity (excluding acral sites)	2	0	2
Foot	27	63	90
Hand	1	7	8
Acral sites (hand and foot)	28	70	98

Twenty-six cases (20%) had metastatic melanoma in the lymph nodes with no known primary sites. 24 cases with lymph node metastasis (92%) were in the inguinal region, suggesting the lower extremity as the possible primary site. In addition, the male sex had a preponderance of metastases (76.9%, *p* value = 0.0001).

### General histologic features

Of 106 melanomas biopsied at the primary site, subclassification was possible in 77 (72.6%). When subclassification was not possible, this was most commonly due to limitations in tumor sampling. For cases that could be subclassified, the most common histopathological subtype was acral lentiginous with 60 cases followed by nodular (11 cases) and superficial spreading (5 cases) (Table 2). There was only 1 case of lentigo maligna. Out of the classifiable 77 cutaneous melanoma cases, 28 were from males. ALM subtype was seen in 23 males (82.1%) and 37 females (75.5%). This difference was, however, not statistically significant, *p* = 0.58.

**Table 2:** Distribution of melanoma subtype and pathological tumour stage (pT) at Kamuzu Central Hospital, Malawi (2012–2017)

pT stage	ALM	NM	SSM	LM	Unclassified	TOTAL
pT1	2	0	0	1	0	3
pT2	2	0	0	0	0	2
pT3	13 (7)	3	1	0	6	23 (7)
pT4	36	8	4	0	20	68
TOTAL	53 (7)	11	5	1	26	96 (7)

Legend: Pathologic tumor stage of melanomas diagnosed at Kamuzu Central Hospital between 2012 and 2017. Numbers in brackets indicate incompletely excised melanomas. pT1, pT2, pT3, pT4 correspond to ≤1.00 mm, 1.01–2.0 mm, 2.01–4.0 mm and > 4.0 mm respectively

Out of 103 cutaneous melanoma cases, 7 were not completely excised and exact Breslow thickness in these cases could not be determined. 68 cases of the 96 cases (70.8%) had Breslow thickness of > 4.00 mm (corresponding to pT4 in the pathologic TNM staging). Tumors were typically biopsied at an advanced stage, with a median Breslow thickness of 9.00 mm. The median Breslow thickness for ALM was 8.5 mm (interquartile range: 5.5–13.5) compared to 7.5 mm (IQR: 4.5–9.5) for the rest of melanoma subtypes excluding the unclassified group. However, this difference was not statistically significant (*p* = 0.42). Similarly, the Clarks level was also assessed in all cutaneous melanoma cases and the most frequent level of invasion for ALM was level IV. Five ALM and ten in the unclassified group were at least Clarks level III.

It was not possible to determine the presence or absence of ulceration in 2 of 103 cutaneous melanoma cases due to the nature of the biopsies. Out of the remaining101 cutaneous melanoma cases, 86 (85%) were ulcerated. The median Breslow thickness of the non-ulcerated cases was 4.5 mm and thus smaller than the median thickness of the all the study cases, however, this is not statistically significant (*p* = 0.077). Nevertheless, the median thickness of non-ulcerated cases remains histopathological stage pT4.

## Discussion

Good health seeking behaviour of women and lack of awareness in males have been proposed as explanations of the female preponderance of melanoma cases in previous studies [4, 5, 14, 22, 28, 31, 32] and similar male to female ratios are identified in our cohort. In addition, a statistically significant increase in metastases occurred in males suggesting delayed presentation.

The incidence of melanoma in Europe and United States of America has shown a plateau or decrease in the younger age groups since the 1980s [15, 18, 19]. Our study however could not infer on incidence as has been done in few studies done in Africa [4, 20]. The data used in this study is not from the population based cancer registry that has a defined denominator needed for calculation of incidence. It is important to emphasize that the Malawian population experiences problems of access (economically and geographically) to centres of modern medical care and shortage of experienced health personnel.

Malignant melanoma affects predominantly adults and elderly patients and according to WHO, peaking around the 6th decade [21]. The protective effects of darker skin in black people reduce the risk of melanoma [21, 23–27] and this may skew the peak to an older age. Our series found the mean age at diagnosis to be 57 years, with a peak in the 7th decade, as reported in Africa and Europe [6, 28, 29]. This may also be attributed to the predominant acral lentiginous melanoma which occurs in an older population than does superficial spreading or nodular melanoma. [6, 33, 34].

In our study 95% of all cutaneous melanomas were located on the acral sites and 87% of these were on the foot, similar to previous reports [4–6, 14, 30, 31, 33, 34]. Both traumatic and genetic explanations have been proposed for this observation. In prior studies, the heel and the inner forefoot have been reported to be most commonly involved [7, 35]. The plantar predilection is thought to be due to repeated trauma and continued pressure on the foot reflecting the less frequent use of shoes, especially in those from rural areas [6, 30, 34, 40–42]. Although genetic predisposition has also been implicated in the predominance of ALM in patients of African ethnicity [9], African-Americans and black Africans are not genetically identical [13]. In addition the impact relative to chronic trauma in low income countries in sub-Saharan Africa has not been thoroughly evaluated. It would be expected that the blacks in African subcontinent have higher incidence of ALM than their African-American counterparts due to additional predisposing factor of chronic trauma. This statistic is however lacking.

Another possibility is that these tumours originate from melanotic naevi and hyperpigmented macules common on plantar surfaces in the black population [34]. The clinical history was not adequate to permit a more specific anatomic evaluation, but cases in the head and neck region (4), trunk (1) thigh (1), knee (1), upper arm (1) and mucosa (3) were identified. This is in contrast to the South African study [5] where they found no melanoma cases above the wrist or ankle. Over 80% of our cases presented with a Breslow thickness of over 4 mm and Clarks level IV, which is similar to other studies from Africa [4, 14]. It has been reported that Clarks level is less reproducible and does not assess prognosis as accurately as tumour thickness, however, it may provide important prognostic information if an accurate Breslow thickness cannot be determined [17].

Ulceration is the second criterion for determining T category. Survival rates of patients with ulcerated melanoma are generally lower than those without of the same T category [16]. The presence of ulceration (85%) and Breslow thickness corresponding to pT4 pathological stage (84%) are both poor prognostic factors which may reflect late presentation and/or diagnosis.

In agreement with studies done in South Africa, Togo, Kenya and Sudan [4–6, 31, 36], our series found acral lentiginous melanoma to be the predominant tumour subtype. However Nigerian and Tanzanian studies [22, 28, 34] found nodular subtype as the predominant subtype, despite the foot being the predominant anatomical site. This is not surprising in that a rapidly growing nodular melanoma can arise within acral lentiginous melanoma and proliferate deeply in the skin. Acral melanomas can also start out as patches with radial growth and then develop a more nodular, vertical growth pattern.

According to WHO [6], acral lentiginous melanoma has a male preponderance and this was also observed in our study series although it was not statistically significant. Acral lentiginous melanoma is known to have a worse prognosis than other melanoma subtypes [6, 8, 38, 39], but controversy still exists as to whether this results from the aggressive biological behavior or delayed presentation [37]. This study did not find a significant statistical difference in Breslow thickness between the two groups (ALM and non-ALM), although both groups had a median value corresponding to pT4 pathological stage.

### Limitations

This study has a few important limitations. First the data in this study is from those who underwent biopsy leaving out those that did not seek medical care and/or were not biopsied. This data may not, therefore, reflect the actual incidence of melanoma in the general population.

Most cases lacked detailed clinical information on the specific location of the tumour precluding comparison to prior publications. For example, most biopsies indicated foot as a primary site without specifying whether this is plantar and/or nail. Although the presence of glabrous skin and nail was helpful in histopathological evaluation, extensive ulceration often obstructed histological features that may have otherwise facilitated more specific localization.

Similarly, detailed clinical and staging information was lacking for many cases, precluding full TNM staging. As such, we were unable to correlate pathologic findings with outcomes in this cohort. In Malawi, there is no Positron Emission Tomography (PET) scan, which would help to more definitively assess for metastasis.

Finally, the availability of only a limited panel of immunohistochemistry (IHC) stains in Malawi prevented confirmation of melanoma in those cases that had focal tumour deposists. In addition, without IHC, it is difficult to definitively distinguish melanoma from other malignancies when the tumor is poorly differentiated. Cases that required IHC for confirmation were therefore excluded in the study, potentially leading to under-diagnosis in our cohort.

## Conclusion

Our cohort of 132 patients is one of the largest Sub-Saharan populations with malignant melanoma investigated within a period of 6 years. Our study shows that malignant melanoma occurs in black people in Malawi and confirms that acral lentiginous melanoma is the predominant subtype, most often occurring on the foot. Further, we show that patients often present with late stage disease associated with poor prognosis. Our findings demonstrate that malignant melanoma is an important cause of death and disability among the Malawian Africans and increased awareness and surveillance is needed to improve prognosis. In addition, these findings suggest that melanoma may be an under-appreciated malignancy in sub-Saharan Africa and our findings emphasize the need for additional descriptions of pathologically confirmed across the region.

## Abbreviations

ALM: Acral lentiginous melanoma
GLOBOCAN: Global cancer Incidence, Mortality and Prevalence
IHC: Immunohistochemistry
IQR: Interquartile range
LMM: Lentigo maligna melanoma
NMM: Nodular malignant melanoma
PET: Positron emission tomography
SSM: Superficial spreading melanoma
TNM: Tumour node metastasis
UICC: Union for international cancer control
